# Social exchange theory tenets and the employment of persons with disabilities in the Western Cape, South Africa

**DOI:** 10.4102/ajod.v15i0.1976

**Published:** 2026-07-15

**Authors:** Warren P. Charles, Tendency Beretu

**Affiliations:** 1 Department of Human Resource Management, Faculty of Business and Management Sciences, Cape Peninsula University of Technology, Cape Town, South Africa; 2 Department of Human Resource Management, College of Economic and Management Sciences, University of South Africa, Pretoria, South Africa

**Keywords:** disability, employment, inclusive practice, public sector, social exchange theory, South Africa

## Abstract

**Background:**

Employment of persons with disabilities (PWDs) in South Africa’s public sector remains persistently low despite progressive legislative frameworks. This reflects enduring structural, organisational and attitudinal barriers that limit meaningful inclusion, particularly at a provincial level. Compliance-driven approaches have proven insufficient in addressing the organisational dynamics shaping inclusion. This study is grounded in the social model of disability and applies social exchange theory (SET) to examine how perceptions of costs, benefits and reciprocity influence employment decisions.

**Objectives:**

This study applied SET to: (1) understand perceived costs/benefits of employing PWDs; (2) explore the role of social structures/actors; (3) propose SET-informed strategies to enhance equity.

**Method:**

A qualitative study, situated within the transformative paradigm and using a case study design, was conducted in the Western Cape provincial public service. Data were collected through semi-structured interviews with 10 senior officials and analysed thematically using key SET principles.

**Results:**

Three interrelated themes emerged: the importance of enabling structures and actors, the role of reinforcement in sustaining inclusive practices, and the significance of reciprocal professional relationships. Findings indicate that inclusion is constrained by perceived costs and limited institutional support, but can be strengthened through structural investment, incentive mechanisms and relational engagement.

**Conclusion:**

Social exchange theory provides a robust framework for reconceptualising PWD employment as a mutually beneficial social exchange, guiding more sustainable, relational inclusion strategies.

**Contribution:**

The study advances SET as a practical lens for designing interventions that address both structural and relational barriers to disability inclusion.

## Introduction

Globally, the integration of persons with disabilities (PWDs) into the labour market remains a persistent challenge central to commitments to equality and social justice (World Health Organization [WHO] 2022). The WHO estimates that over one billion people live with some form of disability, with unemployment rates among PWDs reaching between 50% and 70% in many low- and middle-income countries (Saran, White & Kuper 2020). Across Africa, approximately 75% of working-age PWDs are excluded from the labour force (United Nations Human Rights Council [UNHRC] 2019), underscoring the structural nature of disability exclusion in developing contexts.

In South Africa, this challenge is particularly acute due to intersecting historical and structural factors. The legacy of apartheid systematically excluded Black South Africans with disabilities from education and employment, creating compounded disadvantages that persist despite democratic reforms (Charles 2022). Although the country has adopted progressive legislative and policy frameworks, including the *Employment Equity Act (No. 55 of 1998)* and its ratification of the United Nations Convention on the Rights of Persons with Disabilities (CRPD) in 2007, a substantial gap remains between policy commitments and actual labour market outcomes (Mukwevho & Matlala 2024).

This implementation gap is most visible in the public sector, which has consistently failed to meet its disability employment targets. Despite a long-standing benchmark of 2%, representation of PWDs has remained stagnant at approximately 1% for over two decades (Department of Public Service and Administration [DPSA] 2023; Parliamentary Monitoring Group [PMG] 2025). Recent national data indicate that only 1.05% of the public sector workforce comprises PWDs, with some estimates placing the figure even lower at 0.95% (Republic of South Africa 2025; SABC News 2025). This persistent under-representation exists alongside increasingly ambitious policy targets, including a proposed minimum of 4% for designated employers and a longer-term target of 7% by 2030 (Republic of South Africa 2025). The widening gap between current performance and policy aspirations highlights the limitations of existing approaches to disability inclusion.

At the provincial level, disparities are even more pronounced. In the Western Cape, PWDs constitute only 0.55% of the provincial public service workforce, significantly below both the national average and established policy targets (Republic of South Africa 2025). Representation is also limited at senior levels, with only 1.4% of PWDs occupying top and senior management positions nationally. These patterns suggest that exclusion is not only widespread but also deeply embedded within organisational hierarchies.

Existing evidence points to a range of systemic barriers that continue to hinder the inclusion of PWDs in the workplace. These include discriminatory attitudes, inaccessible physical and organisational environments, inflexible work practices and limited institutional capacity to recruit and retain suitably qualified individuals with disabilities (Republic of South Africa 2025). Collectively, these barriers indicate that the problem extends beyond policy absence to encompass structural and cultural constraints within organisations.

Prevailing approaches to disability inclusion are typically grounded in either human rights or legal compliance frameworks. The human rights approach, informed by the CRPD, emphasises the inherent dignity and equal rights of PWDs, including the right to work on an equal basis with others. In contrast, the legal compliance approach focuses on adherence to statutory requirements, such as employment equity targets and reasonable accommodation provisions. While both approaches are important, they are often insufficient in practice. Compliance-driven strategies, in particular, may encourage a minimalist orientation in which organisations prioritise meeting targets rather than fostering meaningful inclusion.

A key limitation of these approaches is their primary focus on employer obligations and regulatory enforcement, with less attention given to the underlying social and organisational dynamics that shape inclusion. The social model of disability offers an alternative perspective by conceptualising disability as the result of societal and environmental barriers rather than individual impairments (Barnes 2019; Oliver 2013). From this standpoint, improving employment outcomes requires transforming workplace structures, cultures, and practices to remove disabling barriers.

Building on this perspective, there is a need for theoretical frameworks that not only explain exclusion but also account for how inclusive practices are sustained within organisations. This study addresses this gap by integrating the social model of disability with social exchange theory (SET). While the social model explains disability as a product of structural barriers, SET provides a mechanism for understanding how organisational behaviour is shaped by perceived costs, benefits and reciprocity. It suggests that inclusion is more likely to occur when perceived organisational benefits outweigh associated costs and when positive exchanges are reinforced (Darmawan & Gani 2024; Davlembayeva & Alamanos 2025).

Applying SET to disability employment shifts the focus from compliance to mutual value creation. Within the public sector, where performance management systems emphasise measurable outcomes, disability inclusion can be reframed as a beneficial exchange that enhances organisational performance through access to diverse talent, improved innovation, and strengthened social legitimacy. Furthermore, the principles of reciprocity embedded in SET align with the *Batho Pele* [People First] ethos underpinning South African public administration, reinforcing the relevance of this framework in the local context.

Despite extensive policy development, limited research has examined disability employment in the South African public sector through a relational and exchange-based lens. In particular, there is insufficient understanding of how public sector managers perceive the costs and benefits associated with hiring and retaining PWDs, and how these perceptions influence organisational behaviour. Addressing this gap is critical for developing more effective and sustainable inclusion strategies. Despite extensive policy development, limited research has examined disability inclusion in the South African public sector through a relational and exchange-based lens.

Accordingly, this study aims to:

- Critically examine the perceived costs and benefits that influence the willingness of public sector managers to hire and retain PWDs through the lens of SET.
- Explore the role of organisational structures and actors (such as committees and disability champions) in facilitating or constraining disability inclusion.
- Propose a framework of SET-informed strategies to enhance the employment and retention of PWDs in the South African public sector.

### Theoretical underpinnings: Integrating the social model and social exchange

This study is grounded in an integrated theoretical framework that combines the social model of disability with SET to provide both a normative and analytical understanding of disability inclusion in the workplace. The social model of disability serves as the philosophical foundation of the study. It shifts the focus from individual impairments to the societal, environmental, and organisational barriers that restrict participation (Oliver & Barnes 2012; Shakespeare 2006). Within this framework, disability is understood as a socially constructed phenomenon, arising from inaccessible environments, discriminatory practices and exclusionary institutional arrangements rather than inherent limitations of individuals. This perspective is particularly relevant in the South African context, where structural inequalities continue to shape labour market outcomes. Through foregrounding the role of organisational systems and practices, the social model positions employers as key agents responsible for enabling inclusion through workplace transformation (Barnes 2019).

While the social model explains the nature and origin of exclusion, it provides limited insight into how organisations can be motivated to implement and sustain inclusive practices. To address this limitation, this study incorporates SET as an analytical lens. Social exchange theory, originally developed by George Homans (1958) and further advanced by Peter Blau (1964), conceptualises social behaviour as an exchange process in which individuals and organisations evaluate interactions based on perceived costs and benefits (Kitole & Mwita 2025; Ogbonna & Mbah 2022). The theory posits that relationships are maintained when the perceived rewards outweigh the associated costs, and when reciprocal benefits are expected (Wallenburg & Handfield 2022). In organisational contexts, SET has been widely applied to understand employee engagement, organisational commitment and workplace behaviour.

Key principles of SET relevant to this study include:

- **Reciprocity:** Positive actions by one party (such as inclusive hiring practices) are likely to be reciprocated through positive outcomes such as employee commitment, loyalty and performance.
- **Cost–benefit evaluation:** Decision-makers assess the perceived costs (like accommodation, training, perceived risk) against potential benefits (like access to diverse talent, improved organisational reputation, innovation).
- **Reinforcement:** Behaviours that yield positive outcomes are more likely to be repeated, particularly when supported by incentives and recognition systems.
- **Trust and social structures:** Institutional mechanisms such as policies, committees and diversity champions can reduce uncertainty and facilitate sustained positive exchanges.

The relevance of SET to the South African public sector becomes clearer when considered alongside existing governance and performance frameworks. Public sector organisations operate within structured performance management systems that include key performance indicators, employment equity reporting, and compliance scorecards linked to the *Employment Equity Act* (Uzule, Zarina & Shina 2024). These mechanisms are designed to monitor transformation but are often experienced as compliance tools rather than drivers of meaningful inclusion. From a SET perspective, such systems may fail to produce desired outcomes if the perceived costs of employing PWDs outweigh the perceived benefits for managers and organisations (Chumo, Kabaria & Mberu 2023). For instance, if disability inclusion is associated primarily with administrative burden, resource constraints or risk, rather than with organisational value, compliance is likely to be minimal and symbolic. On the other hand, where inclusion is linked to tangible rewards such as improved team performance, enhanced service delivery, innovation or recognition within performance appraisal systems, organisations are more likely to internalise and sustain inclusive practices (Kipara 2025).

Furthermore, the *Batho Pele* [People First] principles, which underpin South African public administration, emphasise service, accountability and responsiveness (Molobela 2024). These principles align closely with the reciprocity norm in SET, as they implicitly frame the relationship between the state and citizens as one of mutual obligation and value exchange. Integrating SET into the analysis, therefore, allows for a more contextually grounded understanding of how inclusion can be embedded within existing public sector values and systems.

Through combining the social model of disability with SET, this study bridges a critical gap in the literature. The social model identifies what needs to change (removal of structural barriers), while SET explains how and why change can occur (through perceived mutual benefit and sustained exchange relationships). This integrated approach moves beyond compliance-based explanations and provides a more dynamic framework for understanding organisational behaviour in relation to disability inclusion.

## Research methods and design

This study was situated within the transformative paradigm, which prioritises social justice, equity and the interrogation of power structures that shape marginalisation (Creswell & Creswell 2018; Korten 2018). This paradigm aligns with the social model of disability by foregrounding structural and institutional barriers as the primary sources of exclusion rather than individual impairment. This study provided an appropriate philosophical foundation for examining how organisational systems and managerial perceptions influence the employment of PWDs in the public sector.

A qualitative case study design was employed to develop an in-depth, context-specific understanding of disability employment practices within the Western Cape provincial public service. The Western Cape Provincial Government Department was selected as a critical case due to its formal obligations under national employment equity legislation and its relevance to public sector transformation.

### Participant sampling and description

Purposive sampling was used to select 10 senior officials occupying Deputy Director and Assistant Director positions within human resource management and policy implementation units. Participants were selected based on their direct involvement in employment equity implementation and disability inclusion decision-making, ensuring alignment with the study objectives.

Access to participants was obtained through formal institutional permission from the relevant provincial department, after which a departmental gatekeeper facilitated initial contact. Eligible participants were invited via official email communication, and participation was entirely voluntary. Inclusion criteria required participants to: (1) hold a senior human resources (HR) or policy implementation role, (2) have direct responsibility for or oversight of employment equity processes, and (3) have a minimum of 2 years’ experience in their current managerial role. All participants provided informed consent prior to data collection. Ethical compliance with the *Protection of Personal Information Act* was strictly observed. Anonymity was ensured through participant codes (P1–P10), and no identifying departmental or personal information was disclosed. Data were securely stored and used solely for research purposes.

### Data collection and analysis

Data were collected through semi-structured interviews conducted between December 2020 and March 2021. Although the data were collected between December 2020 and March 2021, the findings remain relevant given the continued stagnation in disability employment levels in the public sector, which have remained at approximately 1% in recent reports (DPSA 2023; PMG 2025).

Interviews were guided by the central question, ‘How can the employment of persons with disabilities (PWDs) be increased in the South African public sector?’, alongside probing questions exploring organisational practices, perceived barriers and facilitators of inclusion. All interviews were audio-recorded with participant consent and transcribed verbatim to ensure accuracy.

Data were analysed thematically following the six-phase approach outlined by Braun and Clarke (2006). The analysis combined deductive and inductive strategies to ensure both theoretical alignment and sensitivity to participants’ perspectives. In the first phase, transcripts were read repeatedly to achieve familiarisation with the data. This was followed by initial coding, where segments of text were assigned codes reflecting key ideas and patterns.

A deductive coding framework was then applied, guided by the core tenets of SET, namely reciprocity, reinforcement, social structures and cost–benefit exchange. This enabled systematic examination of how organisational behaviours and decisions related to disability inclusion could be interpreted through a social exchange lens. In parallel, inductive coding allowed for the identification of emergent sub-themes not explicitly captured by SET, ensuring that participants’ voices were not constrained by the theoretical framework.

In the third phase, codes were organised into broader categories and candidate themes through constant comparison across participants. These themes were reviewed and refined to ensure internal coherence and clear distinctions between themes. The final themes were defined and named in alignment with both the data and the theoretical framework.

Data saturation was reached after the sixth interview, with subsequent interviews confirming the stability of identified themes. To enhance trustworthiness, the analysis process emphasised credibility through verbatim transcription, consistency in coding, and alignment between data, themes and theoretical interpretation. The resulting themes informed the development of a conceptual model illustrating how structural, relational and reinforcement mechanisms interact to influence the employment of PWDs.

### Ethical considerations

Ethical clearance to conduct this study was obtained from the Cape Peninsula University of Technology Faculty of Commerce Research Ethics Committee (No. 2015FBREC274). It was approved on 25 November 2016. The study followed the general and specific ethical guidelines on informed consent by explaining the study to participants and allowing them to participate voluntarily. The data collected was kept private and confidential, with password protection.

## Results

To contextualise the findings, Table 1 presents the demographic and occupational profiles of participants. The table provides an overview of participants’ positions within the provincial public service and their years of service, which is relevant for interpreting their perspectives on disability inclusion and organisational decision-making.

**TABLE 1:** Participant profile and years in service.

Participant ID	Job title	Gender	Age range (years)	Race	Years in service
P1	Deputy Director	Male	45–54	Black person	13
P2	Assistant Director	Female	35–44	Coloured person	6
P3	Assistant Director	Female	25–34	Coloured person	3
P4	Deputy Director	Female	25–34	Coloured person	2
P5	Assistant Director	Male	45–54	Coloured person	11
P6	Deputy Director	Female	55+	Black person	20
P7	Deputy Director	Male	45–54	Black person	14
P8	Deputy Director	Female	55+	Coloured person	28
P9	Deputy Director	Male	45–54	Black person	12
P10	Deputy Director	Female	55+	Coloured person	30

The profiles presented in Table 1 indicate that participants occupy senior managerial roles with substantial experience in public service, positioning them as key decision-makers in employment equity and disability inclusion processes.

Thematic analysis of the interview data yielded three interrelated themes relating to improving the employment of PWDs in the public sector: (1) the role of enabling structures and actors, (2) the need for reinforcement of inclusive practices, and (3) the importance of reciprocal professional relationships. These themes are presented below with supporting participant excerpts.

### Theme 1: The role of enabling structures and actors

Participants consistently emphasised the need for formal structures and designated roles to support disability inclusion. These included disability management offices, databases of PWDs, and the appointment of disability champions at different levels of government. Such structures were viewed as essential for coordination, accountability and sustained implementation.

One participant highlighted the importance of dedicated roles:

‘There is a need for a disability champion at all levels of government … to conscientise and promote knowledge.’ (P2)

Similarly, another participant emphasised the importance of maintaining systems to facilitate recruitment:

‘Various levels of government should keep a database for PWDs and maintain interaction … to facilitate employment when opportunities arise.’ (P3)

Participants also noted that without such structures, managers often lack the knowledge and confidence to implement inclusion effectively.

### Theme 2: Reinforcement of inclusive practices

A second key theme related to the need to reinforce inclusive behaviour through formal recognition and reward systems. Participants indicated that current approaches focus heavily on compliance, with limited incentives to actively promote disability inclusion.

A participant suggested the introduction of recognition mechanisms:

‘Public Service Departments … should be recognised for preparing to accommodate PWDs … A ranking and recognition system should be devised.’ (P1)

Another participant further emphasised the importance of recognising performance:

‘The performance management system … needs to adequately recognise the performance effort of PWDs. If a PWD performs well, recognise and reward.’ (P6)

These responses indicate that reinforcing positive behaviour both at organisational and individual levels may encourage sustained commitment to inclusion.

### Theme 3: Reciprocal professional relationships

The third theme highlights the importance of respectful, professional engagement with PWDs throughout the employment lifecycle. Participants stressed that inclusion is not only structural but also relational, requiring ongoing interaction, communication and trust.

One participant emphasised the value of social interaction:

‘Promote the social interaction of PWDs among themselves and with others … such exchanges allow PWDs to exchange information that aids their performance.’ (P8)

Another participant linked respect directly to performance outcomes:

‘It is vital for PWDs to be treated with respect … Professional respect of PWDs is likely to translate to their best performance in return.’ (P10)

Participants indicated that such relational dynamics contribute to improved teamwork, communication and overall organisational functioning.

### Summary of findings

Across the three themes, participants highlighted that improving the employment of PWDs requires a combination of structural support, behavioural reinforcement and relational engagement. These findings suggest that inclusion is shaped by both organisational systems and interpersonal dynamics.

**FIGURE 1:**
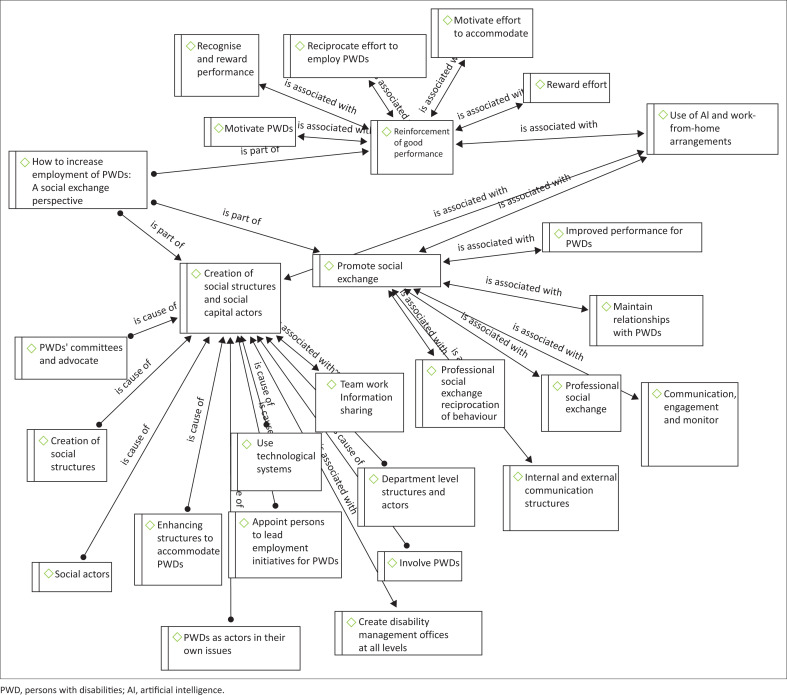
Conceptual framework for increasing employment of persons with disabilities from a social exchange perspective.

A conceptual synthesis of these findings, interpreted through SET, is presented in Figure 1 in the Discussion section.

## Discussion

This study examined the application of SET in understanding and improving the employment of PWDs within the South African public sector. The findings revealed three interrelated themes: enabling structures and actors, reinforcement of inclusive practices and reciprocal professional relationships. Collectively, these themes demonstrate that disability inclusion is not a compliance-driven administrative function, but a dynamic exchange system shaped by perceived costs, benefits and relational obligations within organisational contexts.

Figure 1 illustrates this interactional model, positioning structural, behavioural and relational dimensions as mutually reinforcing components of sustainable inclusion. The model highlights that inclusion emerges through continuous exchanges between organisational systems and individual actors rather than through isolated policy compliance.

### Enabling structures and actors as exchange enablers

The findings indicate that organisational structures such as disability units, dedicated champions and centralised PWD databases function as critical enablers of social exchange. These mechanisms reduce informational asymmetry, clarify organisational expectations and mitigate perceived risks associated with employing PWDs.

Within the logic of SET, such structures lower the transactional costs of inclusion by providing institutional support, thereby increasing the likelihood of positive exchange relationships between management and PWDs. This finding suggests that inclusion is facilitated not only by policy mandates but also through operational systems that actively support decision-making and accountability.

Rather than positioning disability inclusion as an individual adjustment issue, the results reinforce a systemic interpretation in which organisational readiness determines the quality of exchange relationships.

### Reinforcement of inclusive practices as behavioural regulation

The second theme highlights the limited effectiveness of compliance-based approaches in sustaining inclusive behaviour. Participants emphasised the absence of structured incentives and recognition mechanisms for inclusive managerial practices.

From a SET perspective, behaviour is sustained when perceived rewards outweigh perceived costs. The absence of recognition systems weakens the reinforcement loop necessary for sustaining inclusive behaviour among managers and employees.

This finding extends SET by demonstrating that in public sector contexts, inclusion is not only a moral or legal obligation but also a behavioural outcome influenced by organisational reward systems. Embedding disability inclusion within performance management frameworks may therefore strengthen behavioural reinforcement and institutionalise inclusion as a valued organisational outcome.

### Reciprocal professional relationships and relational exchange

The third theme underscores the importance of interpersonal respect, communication and trust between organisational actors and PWDs. Participants reported that respectful treatment fosters commitment, engagement and enhanced performance among PWDs, reinforcing the principle of reciprocity within SET.

These findings demonstrate that inclusion is sustained through ongoing relational exchanges rather than formal structures alone. When employees perceive respect and dignity, they are more likely to reciprocate with loyalty, productivity and organisational commitment.

This relational dimension aligns with broader social capital theory, where trust and interaction enhance organisational effectiveness. In this sense, inclusion becomes both a structural and relational process embedded in everyday workplace interactions.

### Integrated social exchange model of disability inclusion

The three themes collectively demonstrate that disability inclusion operates as an integrated exchange system in which structures enable interaction, reinforcement sustains behaviour and relationships consolidate long-term engagement.

Figure 1 captures this systemic interaction, illustrating that inclusion is not linear but cyclical and mutually reinforcing. This challenges conventional compliance-driven approaches that treat inclusion as a policy obligation rather than an evolving organisational process.

The findings therefore extend SET by demonstrating its applicability in understanding institutional inclusion dynamics within public sector employment contexts in South Africa.

### Theoretical synthesis of participant perspectives

To further strengthen the theoretical interpretation, Table 2 maps participant narratives against SET principles and their organisational implications. This analytical mapping demonstrates how micro-level perceptions reflect macro-level exchange dynamics within the public sector employment environment.

**TABLE 2:** Thematic analysis of participant responses on improving the employment of persons with disabilities.

Participant ID	Key response excerpt (Thematic focus)	SET tenet applied	Implied organisational benefit
P1	‘Public Service Departments … should be recognised for preparing to accommodate PWDs … A ranking and recognition system should be devised.’	Reinforcement (Rewards)	Drives competition and pride in being a leading inclusive employer.
P2	‘There is a need for a disability champion at all levels of government … to conscientise and promote knowledge.’	Social structures/Actors	Reduces managerial uncertainty and provides expert guidance.
P3	‘Various levels of government should keep a database for PWDs and maintain interaction … to facilitate employment when opportunities arise.’	Social structures	Creates a talent pipeline and reduces recruitment costs.
P6	‘The performance management system … needs to adequately recognise the performance effort of PWDs. If a PWD performs well, recognise and reward.’	Reinforcement & reciprocity	Motivates high performance and retains skilled PWD employees.
P8	‘Promote the social interaction of PWDs among themselves and with others … such exchanges allow PWDs to exchange information that aids their performance.’	Social exchange (Trust)	Enhances teamwork, knowledge sharing and organisational social capital.
P10	‘It is vital for PWDs to be treated with respect … Professional respect of PWDs is likely to translate to their best performance in return.’	Reciprocity	Fosters loyalty, commitment and a positive return on investment.

PWD, persons with disabilities; SET, social exchange theory.

Table 2 demonstrates that participants consistently link inclusion outcomes to three core exchange mechanisms: institutional structures (reducing uncertainty and improving coordination), reinforcement systems (driving motivation and recognition) and relational dynamics (building trust and reciprocity).

Importantly, the synthesis shows that perceived organisational benefits such as improved performance, reduced recruitment inefficiencies and enhanced organisational reputation are contingent on the strength of exchange conditions. This reinforces SET’s central proposition that sustained engagement occurs when the exchange relationship is perceived as equitable and beneficial.

Overall, the findings confirm that disability inclusion in the public sector is best understood as a social exchange process shaped by institutional readiness, behavioural reinforcement and relational reciprocity. Through integrating SET with empirical evidence from participants, this study advances a more systemic understanding of inclusive employment practices in South Africa.

### Practical implications

For policymakers and public sector managers, the study suggests a strategic shift from a compliance-centric approach towards a SET-informed relational model of disability inclusion. Practical strategies include:

- **Structural investment:** Mandate and fund the establishment of disability directorates and designated disability champion roles within public sector departments.
- **Incentive design:** Develop and implement performance metrics and reward schemes that recognise and incentivise progress in disability employment, moving beyond compliance-oriented reporting frameworks.
- **Cultural development:** Implement training programmes that build disability confidence among managers and promote social integration initiatives that strengthen trust and social capital between persons with PWDs and non-disabled employees.

### Limitations

This study was conducted as a qualitative case study within a single provincial department; therefore, its findings may not be generalisable across all public sector institutions or provinces. In addition, the sample comprised mainly senior managers, excluding perspectives from frontline human resource practitioners, direct supervisors and PWDs. This results in a predominantly management-centred perspective on disability inclusion.

## Conclusion

This study applied SET to analyse the under-employment of PWDs in the Western Cape public sector. In relation to its objectives, the study identified firstly that perceived organisational costs, such as accommodation uncertainty, often outweigh perceived benefits due to the absence of enabling structures; however, this cost-benefit perception can be reshaped through targeted institutional interventions. Secondly, the study demonstrated that dedicated organisational structures and key actors, such as disability champions and coordinating units, play a critical role in facilitating positive exchange relationships by reducing uncertainty, mitigating risk and providing institutional support. Thirdly, it proposed that reinforcement mechanisms and the cultivation of reciprocal professional relationships are central to sustaining inclusive employment practices over time.

Overall, the findings contribute a SET-informed framework for understanding disability inclusion as a dynamic exchange process rather than a compliance-driven obligation. The significance of this contribution lies in its potential to support the transformation of disability inclusion into a sustained organisational practice grounded in mutual benefit, trust and social value creation. This offers a strengthened conceptual basis for advancing the South African public sector’s equity and inclusion objectives in a more systemic and sustainable manner.
